# Herpes Zoster Infection Culminating in Abdominal Motor Paresis: A Case Report

**DOI:** 10.7759/cureus.100634

**Published:** 2026-01-02

**Authors:** Mohnish Sekar, KavyaDeepu R M

**Affiliations:** 1 Dermatology, Venereology and Leprosy, Karpaga Vinayaga Institiute of Medical Sciences and Research Centre, Chengalpattu, IND; 2 Dermatology, Chettinad Hospital and Research Institute, Chennai, IND

**Keywords:** herpes zoster, motor paresis, phantom hernia, postherpetic neuralgia, pseudohernia

## Abstract

Herpes zoster (HZ) results in numerous complications, the most prevalent being Post Herpetic Neuralgia (PHN). There are fewer than 40 case reports of motor paresis of abdominal muscles secondary to Herpes zoster (Phantom hernia) in the literature to date. The pathogenesis of motor involvement resulting from HZ includes polyneuritis and segmental motor neuropathy, accompanied by subsequent muscle denervation. The viral transmission from the dorsal root ganglion to the anterior horn cells and anterior spinal nerve roots, along with inflammatory involvement of the motor nerve, is believed to contribute to both axonal loss and demyelination. Pseudohernias represent a limited protrusion of the abdominal wall without an evident muscle or aponeurotic defect, secondary to a relaxation of the anterior abdominal wall and resultant bulging because of the intra-abdominal pressure. Here, we report a case of herpes zoster infection involving the T9-T10 dermatome presenting with phantom hernia, which resolved spontaneously within seven months. This case is being reported due to its rarity and highlights the significance of timely and accurate identification to avoid unnecessary evaluation and interventions.

## Introduction

Herpes zoster (HZ) is caused by the reactivation of latent varicella-zoster virus (VZV) within the dorsal root ganglia of the spinal cord, resulting in significant pain and postherpetic neuralgia as its main sequelae. Motor complications can develop when the virus targets the ventral root, specifically affecting motor axons. Around 5% of patients affected with HZ experience motor complications, including Ramsay Hunt syndrome, facial palsy, or segmental limb paresis [1]. Sir W. Broadbent (1866) was the first to document motor involvement following herpes zoster involving the brachial plexus [2]. Followed by Grant and Rowe [3] in 1961 and Thomas and Howard [4] in 1972. The cephalic, thoracic, abdominal, and lumbosacral nerve root segments exhibit motor involvement in descending order. Recently, it has been reported that only 1-5% of patients develop motor neuron involvement of the affected nerves [5]. This case highlights the need for clinical awareness of segmental zoster paresis manifesting as abdominal pseudohernia.

## Case presentation

**Figure 1:**
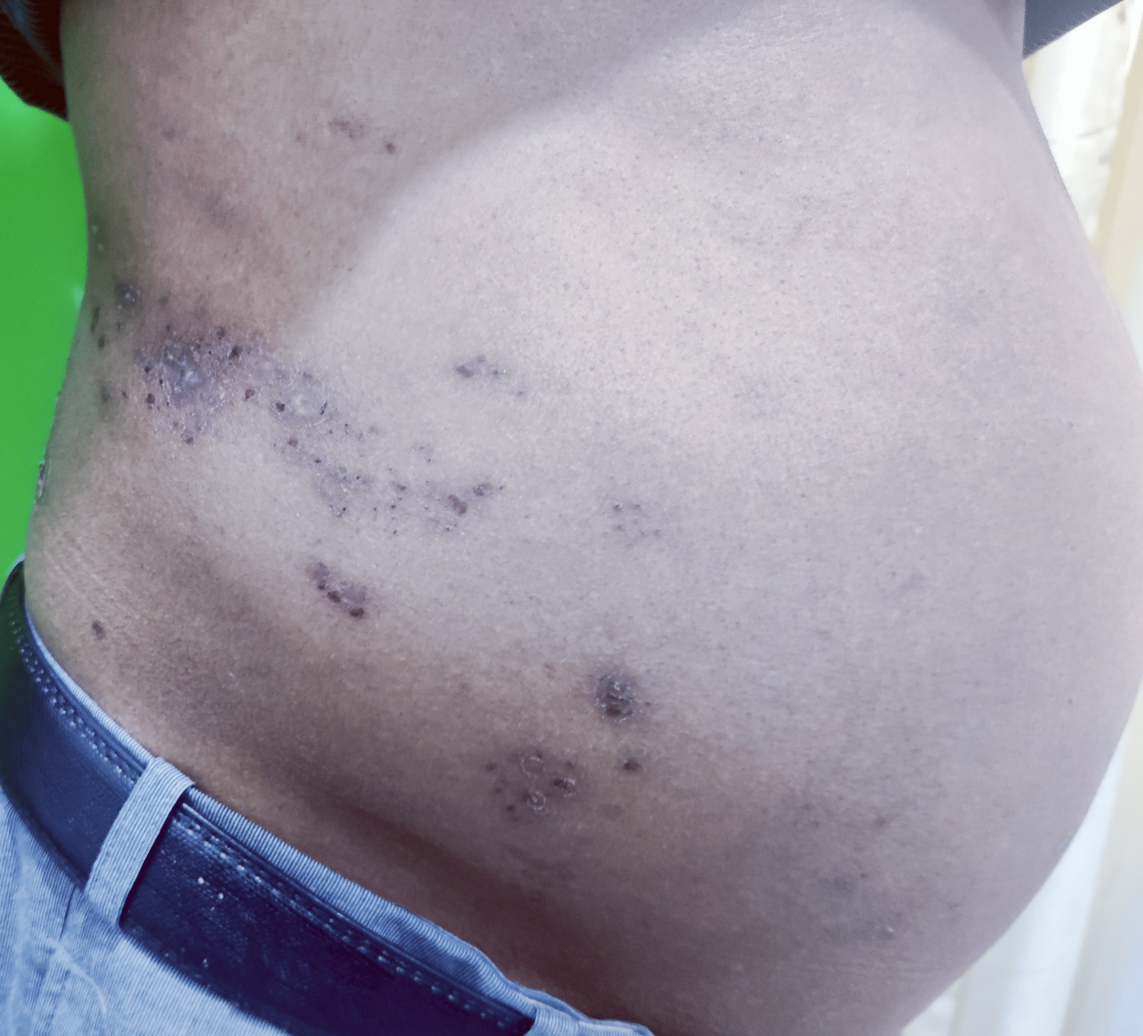
Patient's image on presentation Erythematous to hyperpigmented grouped macules and patches, along with scaling, are observed over the right side of the abdomen, not crossing the midline along the right T9-T10 dermatome.

A 53-year-old male patient presented to us with vesicular eruptions in clusters along the right T9-T10 dermatomes, preceded by pain and dysesthesia for one week. The patient subsequently observed a protrusion on the right side of his abdomen after four days of the crusting stage. The patient is a known case of diabetes (HbA1c 7.2%) and is well controlled with oral hypoglycemic medication. He reported no history of bloating, diminished appetite, or early satiety. No history of pre-existing hernias or surgical intervention in the affected area was noted. Upon clinical examination, erythematous to hyperpigmented macules and patches in clusters were noted over the right side of the abdomen, not crossing the midline, affecting the T9-T10 dermatomes (Figure 1).

**Figure 2:**
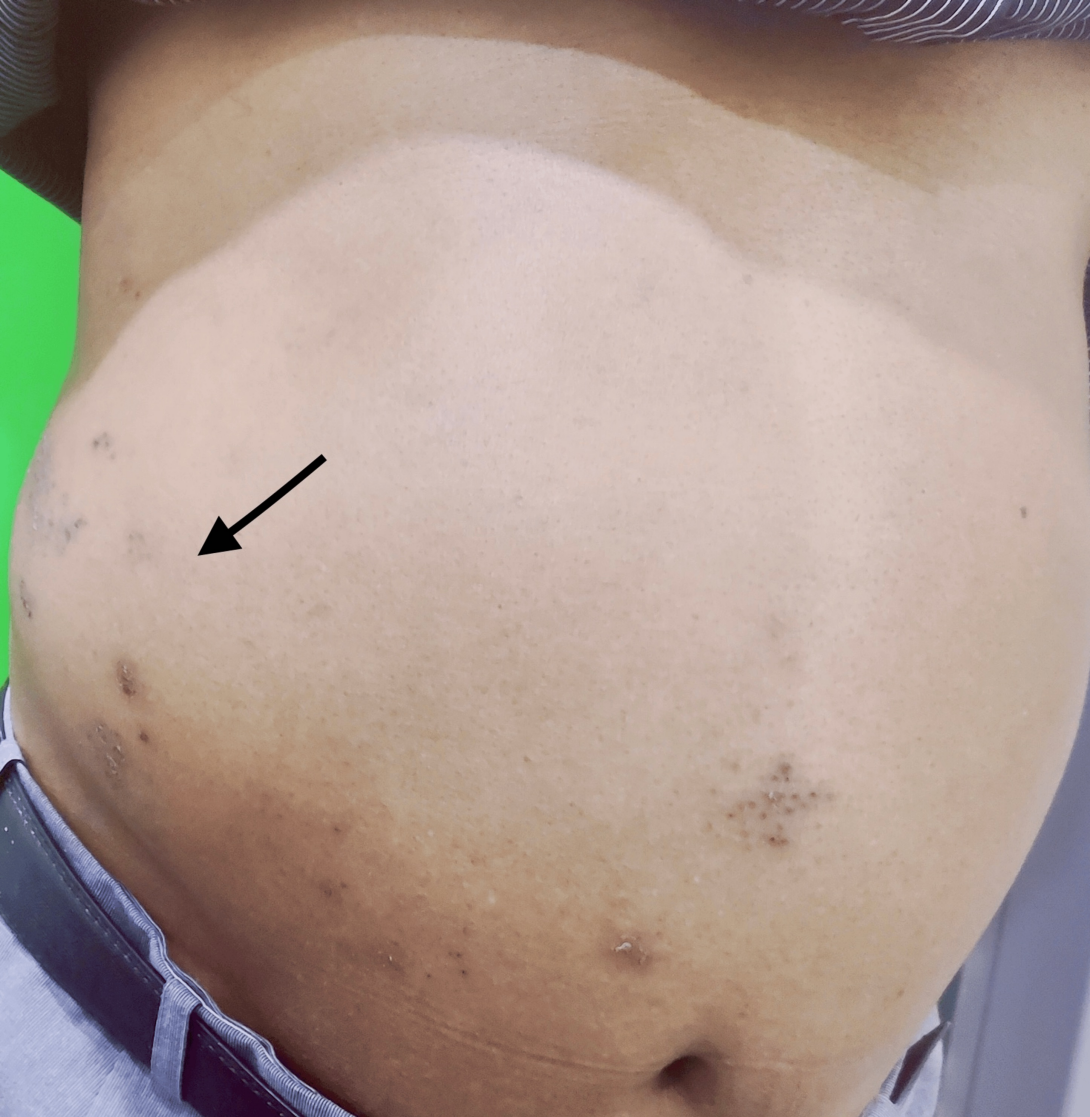
A bulge noted over the right side of abdomen with zoster lesions extending to midline Black arrow denotes the bulge.

A bulge of 10 × 10 cm was observed on the right side of the abdomen (lumbar region) (Figure 2), which became prominent and enlarged when straining, such as coughing and the Valsalva maneuver. No tenderness was detected upon palpation. An abdominal and pelvic ultrasound revealed no abnormality. Differential diagnoses, including incisional hernia, Spigelian hernia, and localized lipoma, were excluded both clinically and radiologically. Characteristic clinical presentation of skin rashes not crossing the midline, which was accompanied by sensory symptoms over the affected dermatome and the absence of abnormalities in the abdomen ultrasonography, a diagnosis of postherpetic pseudo hernia was established. The patient was informed about the self-limiting nature of the condition and was started on pregabalin 75 mg/day, which was continued for five months with symptomatic improvement. The bulge disappeared spontaneously seven months after commencement of the treatment without any consequences.

## Discussion

“Phantom” or “Phantasm” denotes mental imagery generated by imagination. Achar describes a phantom hernia in anterior poliomyelitis as a unilateral abdominal bulge resulting from weakened or paralyzed abdominal wall muscles. A similar type of paralysis that ultimately results in a hernia can also occur in HZ [6]. Pseudohernia, or phantom hernia, originating from herpes zoster (HZ) is also referred to as abdominal wall postherpetic pseudohernia, segmental zoster abdominal paresis, and HZ-induced abdominal paresis. It occurs in about 0.7% of the cases [7]. The majority of cases typically occur within one to eight weeks following the onset of the rash. Additionally, a few cases have been reported prior to the onset of HZ rash [5]. A study by Chernev and Dado stated that the mean age of those affected was 67.5 years, with a male-to-female ratio of 4:1 and the most frequently affected dermatome is T11 [8]. 

Several concepts have been proposed to elucidate the pathogenesis of visceral involvement in HZ, including (1) direct invasion of the varicella-zoster virus (VZV) into the autonomic nervous system of the intestine, (2) vesicular eruption may induce inflammation of the visceral peritoneum, resulting in pain or constipation in the gastrointestinal tract, (3) interaction of autonomic ganglia resulting from the infection of the anterior (ventral) horn of the spinal cord [9]. Typically, abdominal distension resulting from HZ is asymptomatic; however, when the visceral nerve of the gastrointestinal tract is affected, symptoms of pseudo-obstruction of the colon and constipation can develop [1].

A diagnosis of pseudo-hernia resulting from HZ depends on the characteristic clinical presentation of skin rashes not crossing the midline, supported by a prior or current history of HZ, and on protrusion of the abdominal wall that lacks a structural defect. Electromyography (EMG) is employed to identify any nerve conduction abnormalities in the abdominal wall musculature. Additionally, computed tomography (CT) can be utilized to exclude the presence of an abdominal wall hernia caused by a structural defect, fluid accumulation or mass in the affected region [9]. Pseudohernia resulting from HZ typically demonstrates a favourable prognosis, with spontaneous remission occurring after several months. In most documented case reports, symptoms abated spontaneously between three to 12 months following onset [1].

## Conclusions

Abdominal wall pseudohernia should be suspected in patients with recent thoracic HZ presenting with localized bulging without a palpable defect. This protrusion or hernia-like clinical presentation may result from reduced tone of abdominal muscles and fascial architecture, resembling a true hernia, which is profoundly distinct in its pathomechanism. Dermatologists and surgeons must be aware of this entity in order to diagnose patients early, treat them efficiently, and avoid unnecessary diagnostic evaluations.
